# Perceived Factors Influencing Blue-Collar Workers’ Participation in Worksite Health Promotion Programs in Freight Transport: A Qualitative Investigation Using the TDF and COM-B

**DOI:** 10.3390/ijerph21010116

**Published:** 2024-01-21

**Authors:** Marc A. W. Damen, Sarah I. Detaille, Josephine A. Engels, Annet H. De Lange

**Affiliations:** 1 Faculty of Psychology, Open Universiteit, P.O. Box 2960, 6401 DL Heerlen, The Netherlands; 2 School of Organization and Development, Han University of Applied Sciences, P.O. Box 6960, 6503 GL Nijmegen, The Netherlands; 3 Faculty of Social Sciences, Hotel School of Management, University of Stavanger, P.O. Box 8600, 4036 Stavanger, Norway; 4 Department of Psychology, Norwegian University of Science and Technology (NTNU), 7491 Trondheim, Norway; 5 Department of Psychology, Campus de A Coruña, Universidade da Coruña, 15701 A Coruña, Spain

**Keywords:** blue-collar workers, freight transport, worksite health promotion programs (WHPPs), participation, implementation, reach, Theoretical Domains Framework, qualitative research

## Abstract

Blue-collar workers in the freight transport industry report a high risk of developing chronic diseases, partly due to an unhealthy lifestyle. Worksite health promotion programs (WHPPs) may be able to promote a healthier lifestyle, but participation among blue-collar workers in these programs is generally lower than among other workers. The current study aimed to further examine factors that can explain participation of blue-collar workers in these programs. A pragmatic, qualitative study was conducted, and semi-structured interviews were held with 32 blue-collar workers in freight transport in the Netherlands (94% men, 81% driver, mean age 48 (SD = 11)). The interview guide was based on the Theoretical Domains Framework (TDF) and Capability-Opportunity-Motivation-Behavior (COM-B) model, and was used to assess perceived determinants that influence participation. A combination of framework analysis and thematic analysis was conducted, which yielded the following nine main themes: (i) not being aware of WHPPs on offer, (ii) no clear picture of what to expect, (iii) (not) giving priority to health, (iv) expecting feedback and practical support, (v) being open and ready to change, (vi) preferring to be self-dependent, (vii) being offered a practical, fun and joint WHPP, (viii) having an employer who cares, thinks along and facilitates participation, and (ix) working and living in an environment in which a healthy lifestyle is not the norm. With these insights we were able to formulate recommendations to enhance the participation of blue-collar workers in WHPPs.

## 1. Introduction

Blue-collar workers generally have a lower life expectancy, report poorer health and experience more severe health complaints compared with white-collar workers [1,2,3,4,5]. Truck drivers, who make up the largest group of blue-collar workers in freight transport, are no exception. They are at a high risk of developing chronic diseases such as cardiovascular diseases, diabetes, and musculoskeletal disorders [6,7].

An important contributing factor to this increased risk of health problems among blue-collar workers is the unhealthier lifestyle they generally have compared with other workers [8,9,10]. For example, in the US, long-haul truck drivers are twice as likely to smoke and be obese than other workers [11]. Unhealthy lifestyle behaviors have been found to mediate the relationship between occupation and several cardiometabolic diseases [12,13,14]. Therefore, interventions that address unhealthy lifestyle behaviors may improve health and life expectancy among this group.

The workplace is considered a conducive environment for promoting a healthy lifestyle, particularly for hard-to-reach groups [15], as a significant proportion of the population works and spends a substantial portion of their waking hours at work. Recent systematic reviews have shown that Worksite Health Promotion Programs (WHPPs) have significant positive effects on blue-collar workers’ lifestyle and short-term health outcomes [16,17], and are equally effective for blue-collar workers as for white-collar workers [18]. However, existing WHPPs are not very successful in reaching blue-collar workers. They participate in WHPPs less frequently than their white-collar counterparts [19,20,21,22], limiting the potential impact of WHPPs on improving the lifestyle and health of blue-collar workers as a whole.

In the Netherlands, over 123,000 blue-collar workers were employed in freight transport in 2021. Truck drivers, comprising over 90,000 employees, and warehouse workers, numbering over 16,000 employees, are the two largest groups in this sector [23]. The Sector Institute for Transport and Logistics (STL), a collaborative initiative of the industry’s unions and employers’ organizations in the Netherlands, offers a WHPP to all employees of affiliated companies, with the aim of improving the vitality, work ability and general health of employees in the freight transport industry. Some organizations actively encourage participation in this WHPP, while others do not promote it at all. However, all employees of affiliated companies in the sector can participate through the STL website anonymously and free of charge.

The WHPP begins with an online health questionnaire. Based on the questionnaire results, participants are categorized as “at risk” (experiencing health complaints and/or having an unhealthy lifestyle) or “not at risk” (no complaints and a healthy lifestyle). Workers identified as being at risk are invited to participate in a follow-up lifestyle program targeting their specific risks, such as smoking cessation, weight loss, or stress reduction programs. These programs consist of individual live or online sessions with a vitality coach or dietitian, utilizing motivational interviewing, which has been shown to be effective for lifestyle change in previous studies [24,25,26]. In our previous study based on a quasi-experimental effect evaluation, we found a significant positive effect on participants’ vitality, work ability and mental health [27].

Between 2014 and 2021, 12,422 blue-collar workers in the sector completed the health questionnaire provided by STL. Of these workers, nearly 35% were identified as having a health risk. However, only 10.3% of the blue-collar workers with a health risk profile participated in a follow-up intervention [27]. This participation level is even lower than that found in previous studies on WHPP participation among blue-collar workers [28], and it remains unclear what factors influence their participation in the offered WHPP.

Most evaluation research in health promotion focuses on the effectiveness of programs, neglecting the reach and enrollment of participants [29,30,31]. When reach is assessed, researchers often only provide numbers and general characteristics of participants, without providing insight into the underlying factors that influence participation [32].

However, a few studies have investigated factors influencing blue-collar workers’ participation in WHPPs [28]. Quantitative studies have found that intention and self-efficacy to change one’s lifestyle were correlated with participation among blue-collar workers [33,34]. Qualitative studies have explored reasons cited by blue-collar workers for non-participation. Examples of mentioned reasons were: lack of time and opportunity during working hours, concerns about privacy, a negative health culture at work, and not seeing a role for the employer when it comes to lifestyle and health [35,36,37,38,39,40]. Most of these studies assessed determinants without utilizing theory in their design, simply asking workers for reasons for their non-participation [28]. 

This finding aligns with the call from several authors to make more explicit use of theories and theoretical models when studying health behavior [41,42]. To study health behavior, researchers have a plethora of theories and models to choose from, such as the health belief model, theory of planned behavior, or the transtheoretical model of behavior change. However, selecting just one theory or model may result in overlooking key factors that explain behavior [43,44]. The Theoretical Domains Framework (TDF) was developed as an overarching framework to integrate multiple behavior theories [45]. This framework provides researchers with a theoretical lens through which to examine all potential determinants of behavior [46]. 

During the development of the TDF, researchers initially identified 12 domains of determinants from 33 existing theories of behavior or behavior change. Through validation research [45], the model has been refined, resulting in the identification of 14 domains. These domains can be further categorized into the three major components of the Capability-Opportunity-Motivation-Behavior model (COM-B; see Table 1). The TDF and COM-B model have been widely used to investigate barriers and facilitators to health behavior (e.g., [47,48]), including participation in health interventions (e.g., [49,50]).

To the best of our knowledge, no study to date has provided a comprehensive, theory-driven overview of factors that influence blue-collar workers’ participation in WHPPs. Therefore, the aim of this study is to gain a deeper understanding of the determinants that influence blue-collar workers’ participation in WHPPs, using the Theoretical Domains Framework (TDF) and the COM-B model. The main research question of this study is the following: According to blue-collar workers in the freight transport industry in the Netherlands, which factors influence their participation in both health questionnaires and follow-up lifestyle programs as part of a sector-initiated WHPP? Based on the results, we will discuss practical implications for the design and implementation of WHPPs for this target group.

## 2. Materials and Methods

The study followed Atkins et al.’s protocol for using the TDF to investigate implementation problems [46]. The protocol includes seven steps: (1) selecting and specifying the target behavior, (2) selecting the study design, (3) developing study materials, (4) deciding the sampling strategy, (5) collecting the data, (6) analyzing the data, and (7) reporting the findings.

### 2.1. Selecting and Specifying the Target Behavior

The target behavior in this study was blue-collar workers’ participation in a WHPP. Participation was defined as either filling out a worksite health questionnaire or registering for and attending the first lifestyle program session.

### 2.2. Selecting the Study Design 

This study adopted a qualitative approach in which semi-structured interviews were conducted with blue-collar workers in the freight transport industry. The interviews focused on understanding the barriers to and facilitators for participation. As a problem-centered study, it aimed to gain insights into these factors to help find solutions and, therefore, it can be classified within a pragmatic paradigm [51,52,53].

### 2.3. Developing Study Material

The interview guide was developed by two researchers (MD and SD) and consisted of questions for each domain of the TDF, tailored to the specific behavior and context. For example, the domain ‘beliefs about consequences’ was operationalized into the question ‘what did you think the lifestyle program would yield?’ The interview guide was critically reviewed by two vitality experts working for STL and checked and agreed upon by the entire research team (see Supplementary File S1 for the interview guide).

### 2.4. Deciding the Sampling Strategy

Participants in the study were blue-collar workers employed in the freight transport industry in the Netherlands, including truck drivers, couriers, warehouse workers, and crane operators. The inclusion criteria required participants to understand and speak Dutch or English. A quota sampling method was utilized to ensure variation in respondents based on occupation, gender, age, health risk profile, and participation in the WHPP. 

Four methods were employed to recruit participants: telephone, email, HR officers in three freight transport organizations, and advertising in a magazine for truck drivers. Contact details were provided by STL and obtained from the WHPP provider’s database of 12,422 blue-collar workers who had completed the health questionnaire and expressed willingness to participate in future research. Telephone numbers and email addresses were randomly selected from the database. Of the 25 workers approached by telephone, 18 agreed to participate, while six out of the 83 workers approached by email responded positively. Five respondents were recruited through HR officers, and three responded to the magazine advertisement. Participants received a EUR 20 gift voucher as compensation.

Participants who agreed to participate were provided with information about the research, its goals, interview topics, privacy regulations, and data management. A summary of the respondent characteristics is provided in Table 2 (see Supplementary File S2 for all respondent characteristics).

### 2.5. Collecting the Data

Due to COVID-19 lockdown regulations, most of the interviews were conducted over the phone. Participants were either at home or driving while answering interview questions. Face-to-face interviews were held in a separate room at the workplace, with only the researcher and participant present. The interviews were conducted by two researchers (MD and SD) and three student assistants with backgrounds in psychology, and human resource management. All researchers had basic interview skills and, prior to the data collection, discussed the interview guide to ensure agreement on all constructs. All interviews were audio recorded and ranged in duration from 15 min to approximately one hour. 

### 2.6. Analyzing the Data

The interviews were transcribed verbatim and pseudonymized. Following Ojo, et al. [47] and McKeon, et al. [48], a combination of framework analysis and thematic analysis was employed to identify relevant themes in explaining participation. Analysis was conducted using the qualitative data analysis software ATLAS.ti (version 22.2.5.0) for Windows.

First, following Atkins, et al. [46] and Taghy, et al. [54], fragments were deductively coded, using the TDF as an analytical framework, and the 14 TDF domains as the coding categories. Two researchers (MD and SD) performed the initial coding. They coded the same three interviews independently and dilemmas and differences in coding were discussed. Following the protocol by Atkins et al. [46], a guideline for the deductive coding was developed. The coding guideline was discussed and agreed upon in a meeting with the entire research team, and the other 29 interviews were coded (by MD and SD independently).

Following the deductive coding, a thematic analysis was conducted using the steps outlined by Braun and Clarke [55]. This involved familiarizing with the data, initial coding, searching for themes, reviewing themes, and defining and naming themes. Inductive semantic coding was used within each TDF domain to, ultimately, identify main themes related to the three COM-B components (capability, opportunity, and motivation) in explaining participation in the WHPP. All interviews were coded by MD and SD independently. Inductive codes were clustered to form candidate sub-themes, and a thematic map was developed. Perceived factors that influence participation could count as sub-themes, for example, ‘waiting for the right time’, ‘believing that talking won’t solve the problem’, and ‘having privacy concerns.’ It was determined that saturation had been reached when three interviews yielded no new sub-themes. Once saturation was reached, candidate sub-themes were reviewed, defined, and named by MD, SD, and JE. An expert on the TDF (HG) was consulted to resolve any doubts, resulting in a final thematic map with sub-themes per domain. 

Following Patey et al. [56] and Curran et al. [57], a statement capturing the essence of each sub-theme was formulated, and two or three illustrative quotes were selected. Relationships between sub-themes within each COM-B component were analyzed to identify underlying constructs and meanings across the data set, resulting in overarching main themes per COM-B component. These main themes were reviewed, defined, and named by MD and SD. The thematic map with sub-themes and main themes was discussed and agreed upon in the research team. 

### 2.7. Reporting the Results

The consolidated criteria for reporting qualitative studies (COREQ) [58] was used as a checklist (see Supplementary File S3). 

## 3. Results

Eleven TDF domains were found to be relevant in explaining participation in WHPPs. Only the domains ‘reinforcement’, ‘behavioral regulation’, and ‘skills’ were not filled with inductive codes. Each TDF domain was covered by one to eight sub-themes. For example, the TDF domain ‘beliefs about capabilities’ had three sub-themes: ‘believing not being physically able to participate’, ‘believing it will be hard to be open’, and ‘believing not being mentally tough enough to participate.’ (See Supplementary File S4 for all sub-themes per TDF domain.) 

**Figure 1:**
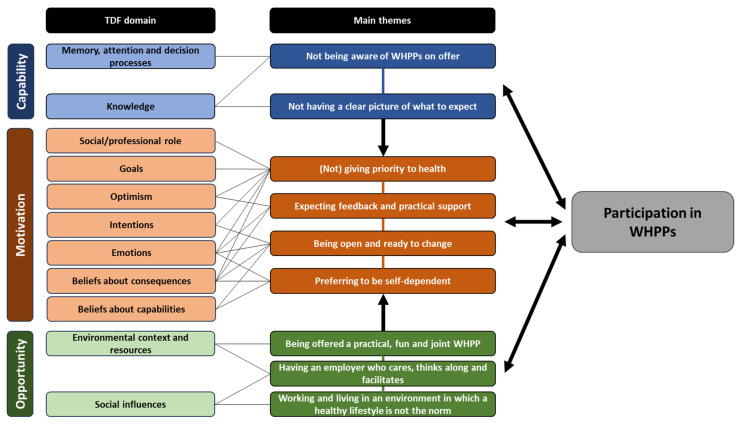
Main themes linked to the TDF and COM-B model as determinants of blue-collar workers’ participation in Worksite Health Promotion Programs (WHPPs).

Nine main themes were identified and agreed upon by the research team (see Figure 1). In this section, we present and describe the main themes. The complete thematic map with sub-themes per TDF domain, corresponding statements, quotes and main themes can be found in Supplementary File S5. 

### 3.1. Not Being Aware of WHPPs on Offer (Capability)

A barrier for participation may be that workers do not receive information about the availability, the content or the registration procedures of the WHPPs. Workers mentioned that they did not receive HRA test results or an invitation to participate in the program. Even when workers do receive information, a barrier may be that they may not notice it or forget to apply or register.

*“I am hardly ever there (at the worksite). The only contact I have is with the planning department. We get a monthly newsletter, but I didn’t see anything about health programs” (respondent 32, man, interstate truck driver, 58 years)*

*“We always have a briefing in the morning (…) The health questionnaire was mentioned a few times and then referred to a few times: did you already do it? It costs nothing, just a bit of your time. It was the second or third time that I thought: oh yeah, I haven’t done that yet” (respondent 18, woman, courier, 41 years)*

### 3.2. Not Having of a Clear Picture of What to Expect (Capability)

A second barrier may be that the information workers receive is too abstract and does not give a clear picture of what to expect. Furthermore, a barrier may be that workers do not know how to apply for participation when WHPPs are not directly offered.

*“I actually only got from it that, hey, we do have certain institutions that could potentially assist you in that, but as for the rest regarding how it works and those kinds of things, no, I haven’t heard anything about that, you know” (respondent 13, man, intrastate truck driver, 39 years)*

*“It wasn’t clear to me where to go—well, I don’t want to participate, but yeah, maybe in the future” (respondent 12, man, truck driver in training, 19 years)*

### 3.3. (Not) Giving Priority to Health (Motivation)

A barrier may be that workers prioritize other urgent matters, such as work–life balance or family issues, over their health. Additionally, everyday hassles may compete with making time for lifestyle changes. For example, workers may prefer to spend their free time with their family instead of going to the gym. Furthermore, workers may underestimate the effects of lifestyle changes or feel like the rewards are not immediate enough, making them less likely to participate in a WHPP.

*“Lack of time. It’s not going to happen. Yeah, the family is on its own then (…) If something takes time, then I say: ‘forget it’” (respondent 24, man, intrastate truck driver, 35 years)*

*“I don’t think that I would feel better if I would eat a few more apples, you see” (respondent 23, man, intrastate truck driver, 52 years)*

Workers mentioned that health becomes more important as they grow older. Sometimes this change in priority comes from observing health deterioration in colleagues or family members of the same age. For others, experiencing health complaints or receiving feedback from a health questionnaire can serve as a wake-up call, making workers aware of the health risks they might face. Becoming a parent or grandparent may also serve as a motivator for participation. The responsibility of being there for their offspring and being able to play with them can motivate them to participate.

*“I took that health check and then it became clear that my cholesterol was way too high (…) That shocked me” (respondent 25, man, interstate driver, 43 years)*

*“And certainly, for the kids. What’s in it for them when they have a fat daddy who is lazy as hell, instead of a fit daddy who can play with them, lives healthy and has a positive attitude towards life?” (respondent 19, man, intrastate driver, 39 years)*

### 3.4. Expecting Feedback and Practical Support (Motivation)

The foremost reason given by workers for participating in WHPPs is the expectation that it will help them improve their health, vitality, or lifestyle. Within the freight transport industry, it can be challenging to maintain a healthy lifestyle, leading some workers to believe that they cannot achieve it on their own. They hope to receive feedback and practical advice on how to combine their work with a healthy lifestyle.

*“For some things, you can try to do it yourself, but if you’re not confident enough, it’s better to have an agency that supports you in some way” (respondent 13, man, intrastate truck driver, 39 years)*

*“I think it’s a good thing, such a health check-up. For all we know, we could be thinking that we’re living very healthily, but then we may have high blood pressure or diabetes. See how many people are dying in the Netherlands, for nothing” (respondent 6, man, intrastate truck driver, 56 years)*

The belief that talking does not solve problems may act as a barrier to participation. Workers mentioned that they are practical and action-oriented. When facing a problem, they think they should address it directly instead of talking about it. This viewpoint makes them skeptical about the potential effectiveness of coaching. If they want to change their lifestyle, they believe they should simply take action.

*“Solving problems by talking, I just don’t always believe in that. I am more of a doer than a talker” (respondent 17, man, intrastate truck driver, 48 years)*

*“Yeah, I had the idea that it would all be a bit ‘woo-woo’, you know? That we would fill in some coloring pages or go searching for a quiet spot in the woods. And that it would all turn out fine […] I thought, ‘that’s not going to help’” (respondent 24, man, interstate truck driver, 57 years)*

### 3.5. Being Open and Ready for Change (Motivation)

Being open to what a WHPP may offer can facilitate participation. Some workers mentioned that they started the WHPP without any preconceived notions to see how it goes. They had no idea what to expect from a WHPP, but they were open to the experience and curious about what a WHPP entails or what may come out of a health test. They began with an open mind and expressed that it does not hurt to try.

*“I just started with an open mind. I had no previous experience with it. I thought, ‘It won’t hurt to talk about it’” (respondent 16, man, intrastate truck driver/mover, 57 years)*

Other workers may be waiting for the right moment to initiate a lifestyle change, convinced that participation is of no use without full commitment. This conviction makes it easy for them to postpone starting a WHPP. They may not be ready to give up life’s pleasures, such as smoking, alcohol, or calorie-rich food, especially when these behaviors serve as coping mechanisms for the stress and difficult working conditions they encounter. They mentioned that there needs to be a turning point that makes them feel “it’s done” before they can commit to a lifestyle change and stick with it.

*“Life just passes by with the way I live and my habits” (respondent 23, man, intrastate truck driver, 52 years)*

*“My wife went through a rough period in the past two years […] and now that everything has settled, she says, ‘It’s okay. It’s been hard for 1.5 years, you know, and now we’re going to enjoy the good things in life’” (respondent 23, man, intrastate truck driver, 52 years)*

### 3.6. Preferring to Be Self-Dependent (Motivation)

Reactance to WHPPs may pose a barrier to participation. Some respondents mentioned that they are accustomed to making their own decisions and do not appreciate others meddling in their personal lives. They believe employers should facilitate a healthy lifestyle, but the decision to participate in WHPPs should be left up to the employee. Furthermore, the idea that a coach will dictate what they should and should not do may not appeal to workers. They may prefer to receive health and lifestyle information in a non-committal manner, such advice via email, giving them the freedom to decide if, where, how, and when they will use the information.

*“No, it’s your employer. It’s all good, but in life, you’re on your own. It’s not up to them. That’s what I think” (respondent 6, man, intrastate and interstate truck driver, 35 years)*

*“If someone says: this is not good and you shouldn’t do that, and you’d better not try that. Yeah, that’s not going to work” (respondent 13, man, intrastate truck driver, 39 years)*

Workers’ skepticism towards the added value of a coach may act as a barrier. They may think a coach is not going to tell them anything they do not already know. Moreover, participants expressed reluctance to share private matters with a stranger, highlighting discomfort with opening up to someone they are not familiar with.

*“Well, I know that I should eat more fruit. So yeah, I don’t need someone, not a dietitian or I don’t know what. That, no, I’ll do it myself, you know” (respondent 17, man, crane operator, 56 years)*

*“Sometimes it’s hard to open up to—so to speak—to…then I think…’well, should I share all this with a stranger, some things?’ you know” (respondent 21, man, intrastate driver, 61 years)*

Another barrier to participation is workers’ concerns about their employer’s intentions and the privacy of their health data. Workers may be skeptical about the anonymity of their health data collected through WHPPs, fearing that it may not be kept confidential. They also worry that their employer could use this information against them, potentially impacting their job security.

*“How anonymous is this all? You’re doing it on the company’s computer. I don’t think it’s all very anonymous” (respondent 12, man, intrastate truck driver, 51 years)*

### 3.7. Being Offered a Practical, Fun and Joint WHPP (Opportunity)

The idea of theoretical and reflective assignments may be seen as burdensome. Instead, workers may prefer practical assignments and engaging activities such as competitions. Offering incentives such as gym membership discounts or healthy meals may also appeal to workers. Furthermore, workers may prefer health checks with feedback and practical advice. They believe that coaches who understand the challenges of adopting and maintaining a healthy lifestyle in the freight transport sector can provide valuable support. Participation will be more likely when a WHPP meets the workers’ preferences.

*“When you’re in the third week of the program, at once you have to do assignment 1, assignment 2 and assignment 3, and that takes like one and a half hour. Well, then you’re quickly fed up with it” (respondent 8, man, interstate truck driver, 42 years)*

*“Then you get to talk to people who have a connection with freight transport, so they know what they’re talking about” (respondent 16, man, 57 years, intrastate truck driver and mover)*

Group activities and personal contact with coaches and other participants are valued, as they provide motivation and help with lifestyle changes. Workers expressed that personal contact is more effective than default solutions or emails.

*“It is the same as a phone call ‘hey, how are you?’ You get more than when you do it with an e-mail. You can easily click an e-mail away” (respondent 20, man, intrastate truck driver, 60 years)*

*“It works best, when you do it as a group, as colleagues. Nobody wants to be in the gym at 6 in the morning, but then you do it anyway” (respondent 18, woman, courier, 41 years)*

### 3.8. Having an Employer Who Cares, Thinks along and Facilitates Participation (Opportunity)

Respondents may appreciate an employer who provides support, arranges and plans health checks, provides information on WHPPs and helps the worker register. This way, the number of steps for participation is minimized. Workers value the convenience of participating during hours that suit them best and at a nearby location. They also appreciate when the costs of participation are covered, as it helps remove financial barriers to participation.

*“Your own general practitioner, yeah, you have to make an appointment and now everything is being planned for you, and you just go” (respondent 4, man, crane operator, 56 years)*

*“I am not going to take some time off to go to a dietician. And that nutrition consultant, well yeah, she made time to come this way on a Saturday” (respondent 10, man, interstate truck driver, 49 years)*

Furthermore, workers respond positively to employers or coaches who show genuine care and interest in their well-being. When employers invest in the well-being of their employees, workers are more willing to participate. While support and facilitation are appreciated, nudging and pressuring may cause reactance. On the other hand, with encouragement, coaches may be able to stimulate participation.

*“You see, it’s an offer from the employer and he sticks his neck out for this, and I’m sure it will cost him something and yeah, I think you should participate then. Right? (…) It works back and forth a bit, you know (…) When they organize a party, I’m there as well” (respondent 23, man, intrastate truck driver, 52 years)*

*“So, the coach called me one day and said she wanted to see me, and I was like: just put some things for me in the e-mail’. But no, she really was like ‘no, I want to see you just once and then we’ll see how it goes from there, but I want to make an appointment’. So she really insisted and, looking back, I think it was good she did” (respondent 20, man, interstate truck driver, 43 years)*

### 3.9. Working and Living in an Environment in Which a Healthy Lifestyle Is Not the Norm (Opportunity)

Negative social norms in the sector may pose a barrier to participation. Workers mentioned that they are being ridiculed by co-workers when they choose to eat a salad or take a walk during their lunch break. Additionally, colleagues may have a negative attitude towards any initiatives introduced by upper management, including the offering of WHPPs. However, many workers mentioned that others’ opinions do not affect their own choices, and they prioritize their own well-being regardless of how others perceive it.

*“Then you’re sitting there with your salad bowl, and they all go like: hey, you’re not a goat, are you?” (respondent 30, man, interstate truck driver, 61 years)*

*“There are a lot of them digging their heels in. They are all like: ‘I don’t want that’. They almost laughed at me. They were like ‘you’re crazy to cooperate’” (respondent 25, man, interstate truck driver, 43 years)*

*“Well, all right, you listen to your colleagues. Everyone has their own opinion and that’s okay. And yeah, then you still think: ‘you choose for yourself and what is best for you’” (respondent 16, man, intrastate truck driver/mover, 57 years)*

At home, partners, in general, agree with participation, but they often do not seem supportive of the lifestyle change. This lack of support can make it difficult for the worker to initiate and maintain lifestyle changes. It may give the worker the impression that they are on their own.

*“My husband is not the type that says: ‘hey, that’s the third time you’re walking to the fridge. Come on, let’s go for a walk outside. I don’t have that kind of support” (respondent 18, woman, courier, 41 years)*

Observing others working on their health can be motivating. Particularly when it comes to sensitive topics such as burnout, personal experiences shared by others may encourage participation. When a worker sees a co-worker who describes similar health issues and acknowledges how a WHPP has aided them, this may serve as a convincing factor for other workers to participate.

*“At a certain point, I thought… there’s a story in this trucker magazine of someone who had gone through the same thing. I thought: ‘that’s going to look a lot like me. Maybe I should fill out such a questionnaire’” (respondent 24, man, interstate truck driver, 57 years)*

## 4. Discussion

Most of our findings are consistent with previous studies on the participation of blue-collar workers in WHPPs. 

Regarding the *capability* to participate, previous studies found that lack of awareness about the program and uncertainty about how to register or what coaching entails were barriers to participation for blue-collar workers [40,59,60,61]. Being on the road, truck drivers mentioned they did not receive information regarding WHPPs. For this reason, drivers are sometimes seen as “hard-to-reach” [40]. Clear communication about the WHPP and its content via multiple, preferably personal, communication channels is recommended to reach this group of workers.

In line with the findings in our study, previous studies found that blue-collar workers may give less priority to health, not feeling the urgency to change their lifestyle when they are feeling healthy [62,63], or they may give priority to other matters, such as spending their little spare time with family [64]. This may lower their *motivation* to participate. Facilitators for motivation that align with previous studies are health risk assessment results serving as a wake-up call [65], and the perceived responsibility to take care of one’s own health as a spouse, parent or grandparent [66]. These facilitators may increase the feeling of urgency to change. To enhance participation, short and simple questionnaires should be considered. Companies can also focus on family values during the recruitment phase (see for example [20]) and begin the WHPP with a health check. To involve workers, it is also recommended that WHPPs take a holistic approach to vitality, integrating lifestyle with safety programs [67] or using a total worker health approach [21,68].

Furthermore, regarding *motivation*, our study revealed that the inclination towards self-reliance may have a negative impact on motivation levels. This finding is in line with previous studies which found that valuing autonomy, the attitude that the workplace should not meddle with personal health matters, and privacy concerns can be barriers to participation for blue-collar workers [37,38,39,40,59,61,66,69].

These findings suggest the importance of involving blue-collar workers in the development and implementation of WHPPs through project groups and co-creation. This approach can ensure that WHPPs meet the needs and preferences of the workers, giving them a sense of ownership and autonomy (see for example [70]). To avoid reactance, it is crucial to clearly communicate that the worker is in charge of the process and can choose whether to participate or comply with advice from coaches or trainers (see also [41]). Addressing privacy concerns may involve outsourcing health questionnaires and individual coaching and clearly communicating how privacy is protected.

Regarding the *opportunity* to participate, we found that a healthy lifestyle can be ridiculed by colleagues in transport companies, which may pose a barrier for WHPP participation. This is consistent with previous research highlighting the perception of weight or health concerns as irrational or unreasonable in many blue-collar work environments [59,71,72]. To address this issue, it is recommended to foster a culture where health is seen as common practice. Strategies such as appointing lifestyle ambassadors, having managers lead by example, discussing health with workers, offering acknowledgement and encouragement, and sharing worker success stories can contribute to creating such a culture. However, employers should be cautious not to push a lifestyle that is too different from the worker’s own. 

In addition, regarding *opportunity*, our research revealed that easy accessibility and flexibility in terms of time and location have the potential to greatly enhance the level of participation. This observation is consistent with previous studies, which have consistently shown that shift work and unfavorable scheduling present significant obstacles to participation [34,66,69,73]. We found that workers highly value employers who actively support and facilitate their participation in WHPPs.

In addition to factors identified in previous studies, this study provided some new insights. Regarding *motivation*, we found that some workers believe that talking about health or health problems does not solve the problem. They may consider themselves doers rather than talkers and may be hesitant to share personal matters with others. Additionally, they may not see the value in talking to a coach and instead believe they should simply change their lifestyle. Therefore, for a portion of this target group, recruitment efforts should be more concrete about what coaching can achieve and include a meeting with the coach in advance. Alternatively, more practical options such as competitions, challenges, gym membership discounts, or healthy lunches should be offered.

Another barrier regarding *motivation* we found is that some workers may be waiting for the right time to begin, and postpone participation. These individuals may not be ready to give up certain pleasures in life. While not being ready for change has previously been identified as a barrier to participation [74], it has also been found that this can, paradoxically, lead to success in lifestyle change through WHPPs [75]. Therefore, it is logical to motivate workers who are not yet ready for change to participate. Furthermore, WHPPs should not overly focus on goal achievement but rather emphasize positive experiences and enhance readiness for change and motivation during the sessions.

Lastly, regarding *opportunity*, having an employer who invests in a healthy work environment was found to be a facilitating factor. The health of the worker should be a shared responsibility, and collective efforts are required to reduce health inequities by reshaping working conditions (see also [76]). To promote participation, it is important for employers to demonstrate their concern for employee health by investing in a healthy work environment. This can help reduce skepticism among workers and encourage them to reciprocate by participating in WHPPs.

One limitation of this study is the underrepresentation of certain subgroups among the respondents. Despite significant efforts to reach non-participants, this particular group proved to be the most difficult to engage. It is possible that the same underlying reluctance to participate in WHPPs also affects non-participation in this study. However, we were able to reach blue-collar workers who started with the health questionnaire, but chose not to participate in lifestyle coaching. Moreover, during interviews, many drivers discussed the social norms within the sector and the opinions they hear from colleagues. In our opinion, this provided sufficient insight into the prevalent attitudes among non-participating blue-collar workers.

In this study, the TDF and COM-B model were found to be valuable in constructing the interview guide. In line with Pinho and Sampaio [41], we emphasize the importance of using holistic, robust and rigorous approaches in studying determinants of behavior in order to guide development and refinement of interventions and implementation strategies in health promotion. The TDF, as a consensus model encompassing 33 theories of behavior and behavior change, helped identify all aspects relevant to explaining participation. We found that this framework was useful in addressing potential blind spots. 

However, it should be noted that the TDF is primarily descriptive and does not capture the interaction or causality between contextual and psychological factors (see also [43]). For instance, a worker who does not feel urgency to participate (Motivation) may forget to register (Capability), requiring reminders (Opportunity). However, workers with a strong sense of autonomy may perceive such reminders (Opportunity) as interference and a threat to their autonomy, leading to reactance (Motivation). Therefore, to attain ontological depth, it seems necessary to combine the TDF with other models or methods. For example, integrating the TDF with a realist evaluation approach could provide further insights into how the context interacts with strategies and personal characteristics to reveal the mechanisms that explain the decision to participate. 

Additionally, it was observed that there was significant variability in the breadth and level of the TDF domains. For example, the domains ‘environmental context and resources’ and ‘social influences’ covered a very wide range of factors, probably reflecting the lack of elaborating context in many behavior theories. Consequently, when employing the TDF to study behavior, the significance of contextual factors may be overshadowed by psychological factors. To more effectively address collective and organizational aspects, it may be advantageous to integrate the TDF with a framework that differentiates among various contextual factors, such as the Consolidated Framework for Implementation Research (CFIR) [77]. 

In contrast, the domain ‘optimism’ was very specific, making it difficult to differentiate from other domains, such as ‘beliefs about consequences’ and ‘emotions’, during the analysis of the interviews. Moreover, TDF domains are derived from both dynamic and static behavior theories, with the result that the domains are not mutually exclusive. As a result, this disparity led to extensive deliberation among the researchers and required a labor-intensive approach to perform the deductive analysis. 

Lastly, to estimate the prevalence of specific beliefs within the target group and the correlation with actual participation, it seems necessary to assess the relevance of domains and codes. Some authors suggest that this can be achieved by examining high frequencies, evidence of strong beliefs, and conflicting beliefs [46,56]. However, in this study, we opted not to categorize specific beliefs as relevant or irrelevant. Instead, through thematic analysis, we integrated all sub-themes into broader main themes. It is possible to use the formulated statements for each sub-theme (see Supplementary File S5) in a survey study to quantitatively explore the prevalence of specific beliefs and their correlation with participation within a specific population or organization.

Nevertheless, using the TDF enabled us to gain insights into the factors influencing participation, and we believe this study offers sufficient starting points for designing WHPPs and implementation strategies for blue-collar workers.

## 5. Conclusions

In this qualitative study, we applied the TDF and COM-B to gain a deeper understanding of the determinants influencing blue-collar workers’ participation in WHPPs in the freight transport industry. Through interviews, we identified nine main themes: (i) not being aware of WHPPs on offer, (ii) no clear picture of what to expect, (iii) (not) giving priority to health, (iv) expecting feedback and practical support, (v) being open and ready to change, (vi) preferring to be self-dependent, (vii) being offered a practical, fun and joint WHPP, (viii) having an employer who cares, thinks along and facilitates participation, and (ix) working and living in an environment in which a healthy lifestyle is not the norm. 

To increase participation, it is recommended that companies invest in creating healthy working conditions and fostering a culture of health. They should consider adopting a holistic approach to health promotion, involving workers in the development and implementation of WHPPs, and removing practical barriers to participation. The promotion of WHPPs could include messages appealing to family values, role models, and emphasis on individual control over one’s own health and lifestyle.

Implementing these strategies may contribute to enhancing the participation of blue-collar workers in WHPPs, ultimately leading to improved employee health and well-being.

## Figures and Tables

**Table 1:** The 14 domains of the Theoretical Domains Framework (TDF) (Retrieved from: Cane, O’Connor, & Michie, Validation of the theoretical domains framework for use in behaviour change and implementation research. Implementation Science, Volume 7, Springer Nature, 2012 [45], Reproduced with permission from SNCSC’).

COM-B Component	TDF Domain
**Capability**	Knowledge
	Behavioral regulation
	Memory, attention and decision processes
	Skills
**Motivation**	Goals
	Intentions
	Social/professional role and identity
	Beliefs about consequences
	Beliefs about capabilities
	Optimism
	Emotion
	Reinforcement
**Opportunity**	Environmental context and resources
	Social influences

**Table 2:** Summary of respondent characteristics.

Variable	Categories	Frequency or Mean
Gender	Men	94%
	Women	6%
Occupation	Driver	81%
	Other	19%
Age		M = 48 years; SD = 11 years
Participation	No participation in the WHPP at all	9%
	Completion of the health questionnaire, but no lifestyle program	28%
	Participation in a lifestyle program	63%

## Data Availability

The data presented in this study are available on request from the corresponding author. The data are not publicly available due to privacy restrictions.

## Supplementary Materials

The following supporting information can be downloaded at: https://www.mdpi.com/article/10.3390/ijerph21010116/s1. S1: Interview guide; S2: Respondent characteristics; S3: COREQ checklist; S4: Sub-themes per TDF domain; S5: Thematic map.

## Abbreviations

COM-B Model	Capability-Opportunity-Motivation-Behavior Model
STL	Sector Institute for Transport and Logistics
TDF	Theoretical Domains Framework
WHPP	Worksite Health Promotion Program
