# Supplementary material for: Perceived Factors Influencing Blue-Collar Workers’ Participation in Worksite Health Promotion Programs in Freight Transport: A Qualitative Investigation Using the TDF and COM-B
Source: Int J Environ Res Public Health. 2024 Jan 21;21(1):116. doi: 10.3390/ijerph21010116 (PMC10815228; doi:10.3390/ijerph21010116)
Supplement: Supplementary file 1 [file ijerph-21-00116-s001.zip › S1 Interview guide.docx]

**Supplementary file S1 Interview guide**

|  | Questions concern participation in worksite health promotion programs (WHPP). Replace WHPP in this interview guide with the specific WHPP that was offered to the respondent, e.g. a health questionnaire or lifestyle coaching |
| --- | --- |
| Introduction | Introduction  Information on research, data management, privacy  Informed consent  Introduction to interview and its topics |
| General | Age  Company  Company size  Job  Education  Years of service  Working Hours per week |
| Knowledge | How did you come to know of the WHPP? What was your first thought?  Were there other ways you heard of the WHPP? If ‘yes’, what were those ways?  What did you know beforehand of the WHPP? How clear was the information you got? Did you receive enough information? Were there things you wish you had known before? If ‘yes’, what were those things? |
| Emotion | How did you feel about participation in the WHPP? |
| memory, attention and decision processes | Where and when did you decide to participate? How did you come to the decision? |
| Beliefs about consequences, goals, and optimism | What were reasons to participate (or not)? What did you hope it would yield? How useful did you think participation would be? What did you see as advantages/disadvantages of participation? |
| Social role and identity | How did the WHPP fit with things that are important to you in life (e.g. values, faith, culture, health, family?) |
| Intentions | Did you immediately know you were going to participate (or not)? How sure were you? |
| Beliefs about capabilities, behavioural regulation, and skills | How hard or easy did you think it would be to participate? What did you think would be hard/easy?  Did you ever start the WHPP? Were there things that made it harder for you to participate? Were there things that made it easier for you to participate? If ‘yes’, what made it harder/easier for you to participate? |
| Social influences | Did you tell people at home you were (not) participating? Why did you (not)? What do you think people around you thought of your (non) participation? Were you being stimulated or were you held back? How were you being stimulated or held back? How did your co-workers react? And your manager? Important people in your home environment? How important are these opinions to you? |
| Environmental context and resources, re-inforcement | How do you feel about your employer offering this WHPP to you? How were you being motivated to participate? Who motivated you? In what form was this done (via letter, e-mail or orally)? What did you think of the way you were being stimulated? What would you have preferred? Were you being facilitated in participation? If ‘yes’, how were you being facilitated? |
