# Supplementary material for: Perceived Factors Influencing Blue-Collar Workers’ Participation in Worksite Health Promotion Programs in Freight Transport: A Qualitative Investigation Using the TDF and COM-B
Source: Int J Environ Res Public Health. 2024 Jan 21;21(1):116. doi: 10.3390/ijerph21010116 (PMC10815228; doi:10.3390/ijerph21010116)
Supplement: Supplementary file 1 [file ijerph-21-00116-s001.zip › S2 Respondent characteristics.docx]

**Supplementary file S2 Respondent Characteristics**

| ID | Occupation | Gender | Age | Participation | Education level | Contract | Company with HR |
| --- | --- | --- | --- | --- | --- | --- | --- |
| 1 | Warehouse worker | Man | 51 | Health questionnaire and coaching, dropped out | Lower secundary | Permanent, full-time | Yes |
| 2 | Intrastate truck driver | Man | 43 | Health questionnaire, no coaching | Upper secondary | Permanent, full-time | Yes |
| 3 | Truck driver in training | Woman | 17 | Health questionnaire, no coaching | Lower secundary | Work/education contract | Not sure |
| 4 | Crane operator | Man | 56 | Health questionnaire, no coaching | Unknown | Permanent, part-time | yes |
| 5 | Intrastate truck driver | Man | 39 | Health questionnaire and coaching, dropped out | Early childhood education | Permanent, full-time | Yes |
| 6 | Intrastate truck driver | Man | 56 | Health questionnaire, no coaching | Unknown | Temporary, full-time | Not sure |
| 7 | Intrastate truck driver | Man | 48 | Health questionnaire and coaching, dropped out | Lower secondary | Permanent, full-time | Yes |
| 8 | Interstate truck driver | Man | 42 | Health questionnaire and coaching, dropped out | Upper secondary | Temporary, varies | Agency |
| 9 | Intrastate truck driver | Man | 53 | Health questionnaire and coaching, completed | Upper secondary | Permanent, full-time | Yes |
| 10 | Interstate truck driver | Man | 49 | Health questionnaire and coaching, dropped out | Early childhood education | Permanent, full-time | Not sure |
| 11 | Forklift driver | Man | 52 | Health questionnaire, no coaching | Unknown | Permanent, full-time | Yes |
| 12 | Truck driver in training | Man | 19 | Health questionnaire, no coaching | Lower secondary | Work/education contract | Not sure |
| 13 | Intrastate truck driver | Man | 39 | Health questionnaire and coaching, completed | Upper secondary | Permanent, full-time | Yes |
| 14 | Intra- and interstate truck driver | Man | 35 | Health questionnaire and coaching, dropped out | Unknown | Permanent, full-time | Not sure |
| 15 | Intrastate truck driver | Man | 52 | Health questionnaire and coaching, completed | Unknown | Permanent, full-time | Yes |
| 16 | Intrastate truck driver and mover | Man | 57 | Health questionnaire and coaching, completed | Unknown | Permanent, full-time | Not sure |
| 17 | Intrastate truck driver | Man | 48 | Health questionnaire and coaching, completed | Upper secondary | Temporary, full-time | Yes |
| 18 | Courier | Woman | 41 | Health questionnaire and coaching, completed | Upper secondary | Permanent, part-time | Yes |
| 19 | Intrastate truck driver | Man | 51 | Health questionnaire and coaching, completed | Lower secondary | Permanent, full-time | Yes |
| 20 | Intrastate truck driver | Man | 60 | Health questionnaire and coaching, completed | Unknown | Unknown | Unknown |
| 21 | Intrastate truck driver | Man | 61 | Health questionnaire and coaching, completed | Lower secondary | Permanent, full-time | Yes |
| 22 | Interstate truck driver | Man | 52 | Health questionnaire and coaching, completed | Lower secondary | Permanent, full-time | Yes |
| 23 | Intrastate truck driver | Man | 52 | Health questionnaire, no coaching | Lower secondary | Permanent, full-time | Yes |
| 24 | Intrastate truck driver | Man | 35 | Health questionnaire, no coaching | Upper secondary | Permanent, full-time | Yes |
| 25 | Interstate truck driver | Man | 43 | Health questionnaire and coaching, dropped out | Upper secondary | Permanent, full-time | Yes |
| 26 | Interstate truck driver | Man | 55 | Health questionnaire, no coaching | Lower secondary | Permanent, full-time | Yes |
| 27 | Interstate truck driver | Man | 57 | Health questionnaire and coaching, completed | Lower secondary | Permanent, full-time | Yes |
| 28 | Interstate truck driver | Man | 57 | Health questionnaire and coaching, completed | Unknown | Unknown | Unknown |
| 29 | Crane operator | Man | 54 | Health questionnaire and coaching, completed | Lower secondary | Permanent, full-time | Yes |
| 30 | Interstate truck driver | Man | 61 | No health questionnaire, no coaching | Unknown | Unknown | No |
| 31 | Interstate truck driver | Man | 50 | No health questionnaire, no coaching | Lower secondary | Permanent, full-time | No |
| 32 | Interstate truck driver | Man | 58 | No health questionnaire, no coaching | Upper secondary | Permanent, unknown | Yes |
