# Supplementary material for: Perceived Factors Influencing Blue-Collar Workers’ Participation in Worksite Health Promotion Programs in Freight Transport: A Qualitative Investigation Using the TDF and COM-B
Source: Int J Environ Res Public Health. 2024 Jan 21;21(1):116. doi: 10.3390/ijerph21010116 (PMC10815228; doi:10.3390/ijerph21010116)
Supplement: Supplementary file 1 [file ijerph-21-00116-s001.zip › S4 Sub-themes per TDF-domain.docx]

**Supplementary file S4.** Sub-themes per TDF domain and Capability-Opportunity-Motivation-Behavior (COM-B) Component

| COM-B Component | TDF Domain | Sub-theme | Number of fragments |
| --- | --- | --- | --- |
| Capability | Knowledge | Barrier: Missing information about the WHPP | 20 |
|  |  | Barrier: Not having a clear picture of the WHPP | 38 |
|  |  | Barrier: Not knowing how to apply | 9 |
|  | Memory, attention and decision processes | Barrier: Forgetting to apply | 16 |
| Motivation | Goals | Facilitator: Matching WHPP with own health goals | 17 |
|  |  | Barrier: Having other priorities | 12 |
|  |  | Facilitator: Finding mental and/or physical health important | 9 |
|  | Social/ professional role | Facilitator: Feeling responsibility towards family | 7 |
|  |  | Facilitator: Feeling responsibility towards work | 14 |
|  | Intentions | Facilitator: Feeling the need to change the way of living | 22 |
|  |  | Barrier: feeling no need to change the way of living | 8 |
|  |  | Barrier: Waiting for the right time | 27 |
|  |  | Facilitator: Already involved in health activities | 15 |
|  |  | Facilitator: Not overthinking participation too much | 35 |
|  | Optimism | Facilitator: Being hopeful | 8 |
|  |  | Facilitator: being aware of health risks | 12 |
|  |  | Barrier: not being aware of health risks | 5 |
|  | Emotions | Facilitator: Feeling enthusiastic | 4 |
|  |  | Facilitator: Being concerned | 2 |
|  |  | Facilitator: Being curious | 11 |
|  |  | Barrier: Being skeptic | 15 |
|  |  | Barrier: Feeling reactance | 12 |
|  | Beliefs about consequences | Facilitator: Believing the WHPP will improve health | 50 |
|  |  | Barrier: Believing a coach will tell nothing new | 10 |
|  |  | Facilitator: Believing a coach can offer support | 10 |
|  |  | Barrier: Believing that talking won’t solve the problem | 8 |
|  |  | Barrier: Believing you have to give up individual freedom | 13 |
|  |  | Barrier: Having privacy concerns | 11 |
|  |  | Facilitator: Expecting regret for not participating in the future | 4 |
|  |  | Barrier: Believing you have to give up on pleasures in life | 13 |
|  | Beliefs about capabilities | Barrier: Believing not being physically able to participate | 5 |
|  |  | Barrier: Believing it will be hard to be open | 12 |
|  |  | Barrier: Believing not being mental tough enough to participate | 17 |
| Opportunity | Environmental context and resources | Barrier: Facing complex or inconvenient procedures for participation | 12 |
|  |  | Facilitator: Not having to arrange things yourself | 28 |
|  |  | Facilitator: Having a say in where and when the WHPP takes place | 22 |
|  |  | Facilitator: Being offered practical and fun activities | 17 |
|  |  | Facilitator: Being offered activities with personal contact | 19 |
|  |  | Facilitator: Being offered a WHPP that is adjusted to the sector/job | 11 |
|  | Social influences | Barrier: Experiencing a lack of support and care from employer | 23 |
|  |  | Facilitator: Experiencing support and care from employer or coach | 34 |
|  |  | Barrier: Experiencing lack of support at home | 35 |
|  |  | Barrier: Working in an environment in which behaving healthy is being ridiculed | 48 |
|  |  | Barrier: Working in an environment in which colleagues are negative about staff or upper management initiatives | 9 |
|  |  | Facilitator: Hearing personal stories and seeing role models | 9 |
|  |  | Facilitator: not caring about others’ opinions | 36 |
